# Corrigendum

**DOI:** 10.2471/BLT.15.100415

**Published:** 2015-04-01

In Volume 86, Issue 4, April 2008, page 260, the third sentence of the first paragraph should begin: “In 2000, it was estimated that over 21.6 million episodes of typhoid occurred worldwide…”.

